# *Fusobacterium nucleatum* promotes the development of acute liver failure by inhibiting the NAD^+^ salvage metabolic pathway

**DOI:** 10.1186/s13099-022-00503-2

**Published:** 2022-06-28

**Authors:** Pan Cao, Qian Chen, Chunxia Shi, Luwen Wang, Zuojiong Gong

**Affiliations:** Department of Infectious Diseases, Renmin Hospital of Wuhan University, 238 Jie Fang Road, Wuhan, 430060 People’s Republic of China

**Keywords:** Acute liver failure, Energy metabolism, Gut-liver axis, *Fusobacterium nucleatum*, Inflammation

## Abstract

**Background:**

Acute liver failure (ALF) patients are often accompanied by severe energy metabolism abnormalities and intestinal microecological imbalance. The intestinal mucosal barrier is severely damaged. Intestinal endotoxin can induce intestinal endotoxemia through the "Gut-Liver axis". More and more evidence shows that members of the gut microbiota, especially *Fusobacterium nucleatum* (*F. nucleatum*), are related to inflammatory bowel disease, but whether *F. nucleatum* is involved in the development of ALF and whether it affects the liver energy metabolism is unclear.

**Methods:**

This study first detected the abundance of *F. nucleatum* and its effect on ALF disease, and explored whether *F. nucleatum* aggravated liver inflammation in vitro and in vivo.

**Results:**

Our data showed that liver tissues of ALF patients contained different abundances of *F. nucleatum*, which were related to the degree of liver inflammation. In addition, we found that *F. nucleatum* infection affected the energy metabolism of the liver during the development of ALF, inhibited the synthesis pathway of nicotinamide adenine dinucleotide (NAD^+^)'s salvage metabolism, and promoted inflammatory damage in the liver. In terms of mechanism, *F. nucleatum* inhibited NAD^+^ and the NAD^+^-dependent SIRT1/AMPK signaling pathway, and promoted liver damage of ALF.

**Conclusions:**

*Fusobacterium nucleatum* coordinates a molecular network including NAD^+^ and SIRT1 to control the progress of ALF. Detection and targeting of *F. nucleatum* and its related pathways may provide valuable insights for the treatment of ALF.

**Supplementary Information:**

The online version contains supplementary material available at 10.1186/s13099-022-00503-2.

## Background

Acute liver failure (ALF) is a clinically critical illness that causes severe disorders of liver synthesis, detoxification, and metabolism due to multiple factors. Its pathological manifestations are massive necrosis of liver cells, accompanied by severe degeneration of viable liver cells [1]. At present, medical treatment for ALF is limited to etiological treatment and symptomatic treatment, and there is still no effective treatment for its pathogenesis [2].

The liver is the central organ of human nutrient metabolism, and its abnormal function can be accompanied by severe energy metabolism disorders. The mitochondria of hepatocytes, as the main place of the tricarboxylic acid cycle [2], which provides sufficient energy for the metabolism and function of the liver [3]. Patients with ALF are often accompanied by severe energy metabolism abnormalities and intestinal microecological imbalances. The intestinal mucosal barrier is severely damaged, and intestinal endotoxin can induce intestinal endotoxemia through the "Gut-Liver axis" [4]. Endotoxin derived from the intestine can not only directly destroy liver tissues, but also induce local non-specific hypersensitivity reactions in the liver, induce hepatic macrophages to release a large number of cytokines, and further produce a natural immune cascade reaction, resulting in a "second blow", and aggravated the damage of liver cell during ALF [5]. Liver failure is accompanied by severe abnormal energy metabolism. Intestinal endotoxemia damages the energy metabolism of liver cells and may be an important factor in the occurrence of liver failure [4]. Studies have shown that endotoxin can directly damage the mitochondria of liver cells [6], disrupt the tricarboxylic acid cycle, and induce liver cell apoptosis [5]. Therefore, how to reduce the damage of endotoxin to liver cell energy metabolism may provide new ideas for the clinical treatment of liver failure.

Nicotinamide adenine dinucleotide (NAD^+^) is an important coenzyme in the process of biological metabolism. It plays an indispensable cofactor in mitochondrial oxidative phosphorylation and participates in oxidative phosphorylation and redox reactions, DNA repair and inflammation [7, 8]. The main mechanism for maintaining NAD^+^ levels in mammalian cells is the salvage synthesis pathway, accounting for 85% of human NAD^+^ sources. In this pathway, Nicotinamide (NAM), as the precursor of NAD^+^, is converted into nicotinamide mononucleotide (NMN) under the action of nicotinamide phosphoribosyl transferase (NAMPT) and then converted for NAD^+^ [9]. Recent studies have shown that an important feature of aging is the reduction of NAD^+^ level [10, 11]. In animal models, supplementation of NAD^+^ can also treat age-related fatty liver and diabetes [12]. There is evidence that the mammalian NAD^+^ salvage synthesis pathway plays a key role in mediating bacteria and NAD^+^ homeostasis[8], indicating that NAD^+^ may be involved in the signal exchange between the host and the bacteria. However, the specific mechanism between the reduction of NAD^+^ and the development of ALF is still rarely reported.

In recent years, the intestinal flora has played a key role in colitis [13]. The balance of the intestinal flora is very important to the stability of the intestinal immune system. The imbalance of the flora will trigger an excessive intestinal immune response, and in severe cases, it will lead to the destruction of the intestinal mucosal barrier [14]. *Fusobacterium nucleatum* (*F. nucleatum*) has been found to cause opportunistic infections for a long time and promote the apoptosis of human gingival fibroblast lymphocytes. In recent years, it has been considered to be related to colitis and colorectal cancer and has been found in many patient tissues and can promote the progression of the disease [15]. A complete intestinal mucosal barrier is essential to prevent toxins and antigens from entering the immune cells [16]. Studies have shown that after the bacterial biofilm of *F. nucleatum* and cells contact incubation, the bacteria can penetrate the basement membrane barrier and invade the collagen matrix [17]. In patients with colitis and colorectal cancer, *F. nucleatum* can invade the mucosa and destroy the mucosal barrier function [18, 19]. Once the homeostasis of the intestinal environment is destroyed, the intestinal flora and its products will enter the liver through hepatoenteric circulation, causing a series of immune and inflammatory reactions in the liver. However, whether *F. nucleatum* can affect the progression of ALF through the "Gut-Liver axis" is unclear.

In this study, we tried to explore the exact relationship between energy metabolism and intestinal flora in the occurrence and development of liver inflammation. We confirmed the reduction of liver NAD^+^ content in ALF and the invasion of *F. nucleatum* might promote the progression of ALF, and found that NAMPT-mediated NAD^+^ salvage synthesis pathway was impaired, while the indolamine 2,3-dioxygenase (IDO)-mediated NAD^+^ de novo synthesis pathway was not impaired, Besides, *F. nucleatum* inhibited the expression of SIRT1 through the NAMPT/NAD^+^ pathway, aggravating liver damage.

## Materials and methods

### Clinical samples

The liver tissues of ALF patients used in this study came from patients undergoing liver transplantation at the Renmin Hospital of Wuhan University (Hubei, China). Normal tissue biopsies were obtained from the donor liver in liver transplantation. The project was approved by the institutional review board and all participants provided informed consent (Approve number: 2021-K016). The patients were screened and excluded including infectious diarrhea, primary sclerosing cholangitis, recent malignant tumors, history of hormone or immunosuppressive therapy and flora transplantation in the past two years, and those who have used antibiotics in the past six months. Formalin-fixed and paraffin-embedded liver tissues were used for fluorescence in situ hybridization (FISH) analysis and immunohistochemical staining.

### Liquid chromatography–mass spectrometry (LC–MS) metabolomics

For liver tissue samples, mixed 20 mg of tissue with 200 μL of pre-chilled water and 800 μL of pre-chilled methanol/acetonitrile (1:1, v/v). Centrifuged at 16000 g for 20 min at 4 °C. Added the same amount of internal standard L-Glutamate-d5 to each sample and dried it in vacuum. And then added 100 μL of acetonitrile-water solution (1:1, v/v) for reconstitution, centrifuged at 16000 g at 4 °C for 15 min, and taked the supernatant for analysis. Shimadzu Nexera X2 LC-30AD high performance liquid chromatography was used for separation. All materials were purchased from Sigma-Aldrich or Shanghai Yuanye Biology; Acetonitrile (Millipore, 1.00030.4008), methanol (Millipore, 1.06007.4008), formic acid (Fluka, 06,450), ammonium acetate (Sigma, 70,221).

### Bacterial strains and cell culture

The human liver cell line L02 obtained from China Center for Type Culture Collection (CCTCC) were cultured in DMEM medium (HyClone, USA) containing 10% heat-inactivated FBS (GIBCO, USA) and 100 U penicillin/100 g streptomycin (Sigma, USA) at CO_2_ atmosphere with a temperature of 37 °C and a humidity of 5%. TNF-α (100 ng/mL, Sigma, USA) combined with D-Galactosamine (D-Gal, 44 μg/mL, Sigma, USA) were used to establish the ALF model in vitro. Constructed NAMPT overexpression plasmid and enveloped it with lentiviral vectors (LV, GeneCreate, China). Besides, the cells were treated with the fresh media containing 1 mM AMPK activator 5-aminoimidazole-4-carboxamide-1-b-D-riboside (AICAR). After 24 h treatment, the cells were harvested for further experiments. *Fusobacterium nucleatum* (ATCC10953, Beijing, China) were cultured on tryptic soy under anaerobic conditions. *Escherichia coli* (*E.coli*) strain (Beijing, China) were cultured in Luria-Bertani (LB) medium.

### Mice

Male C57BL/6 J wild-type mice aged 5–6 weeks purchased from Wuhan Biomedical Research Institute were raised in the specific pathogen free (SPF) animal facility of Renmin Hospital of Wuhan University under conditions of light-controlled, room temperature 25 °C, humidity 55 ± 5% and they were free to eat and drink. The laboratory animal facility use license number was No. SYXK (Hubei) 2015–0027. The operations were approved by the Animal Care and Use Committee of Renmin Hospital of Wuhan University, China.

### Animal models

All mice were randomly divided into nine treatment groups with 6 mice in each group: saline control group; ALF group; *E.coli* group; *F. nucleatum* group; *E.coli* + ALF group; *F. nucleatum* + ALF group; *F. nucleatum* + ALF + Ad-lacZ group; *F. nucleatum* + ALF + Ad-SIRT1 group and *F. nucleatum* + ALF + Nicotinamide Riboside (NR) group. All mice were given 2 mg/ml streptomycin in drinking water for 3 days. After that, PBS-resuspended *F. nucleatum* (10^9^ CFU/ml) or PBS alone was administered to mice by gavage every day for 4 weeks [20]. In NR supplement group, NR was administered to mice by gavage every day for 4 weeks [21]. The adenovirus overexpressing mouse SIRT1 was prepared with RAPAd® adenovirus expression system (Cell Biolabs, Inc) and purified to 10^11^ PFU. Ad-LacZ (control, 50 μl) and Ad-SIRT1 (50 μl) were injected to mice once a week. Intraperitoneal injection of LPS (100 μg/kg, L2880; Sigma) and D-gal (400 mg/kg, G0050; Sigma) [22] was used to establish the ALF model after NR and *F. nucleatum* pretreatment for 4 weeks. Animals were quickly euthanized at 24 h time point after D-gal and LPS administration and blood samples and liver tissues were harvested.

### Fluorescence in situ hybridization (FISH)

Performed microbial FISH as described [18]. Prepared 5-µm-thick sections and hybridized by using commercial kits (FOCOFISH, Guangzhou, China). The sequence of the probe were as follows: 5′-GCT GCC TCC CGT AGG AGT-3′ for “universal bacterial” probe (EUB338; Cy3 labeled); 5′-CTT GTA GTT CCG C(C/T) TAC CTC-3′ for *F. nucleatum*-targeted probe (FUS664; FITC labeled). Slides were examined using a microscope (BX53F; Olympus, Tokyo, Japan).

### Histopathological examination

Fresh liver tissue was fixed with paraformaldehyde for 24 h. The liver tissues were sliced completely and then stained with haematoxylin–eosin (HE). Light microscope (Olympus, Japan) was used to observe and evaluate the pathological changes of liver tissues. The liver histology score was used to judge the degree of liver damage in the ALF model, including inflammation and necrosis scores [23].

### Immunohistochemical staining and immunofluorescent staining

The liver tissues were sliced completely and the thickness was 4–6 μm and uniform. Drop the cell suspension of L02 cells onto the cover glass and place it in an incubator with a CO_2_ concentration of 5% at 37 °C until the cells are fixed (about 2 h). Sections were dewaxed, incubated with 3% H_2_O_2_, blocking serum and thereafter incubated with a 1:100 dilution of polyclonal antibodies against TNF-α; IL-1β; F4/80 or CD68 (Santa Cruz Biotechnologies, CA, USA). Slides were imaged using light microscope (Nikon) and fluorescent microscope (Olympus, Japan).

### Biochemical analyses and detection of NAD^+^

Hitachi Automatic Analyzer (Japan) was used to detect the levels of alanine aminotransferase (ALT) and aspartate aminotransferase (AST). Commercial kits (Nanjing Jiancheng Bioengineering Institute, Nanjing, China) were used to detect the levels of superoxide dismutase (SOD), malondialdehyde (MDA) and glutathione peroxidase (GSH-Px). The levels of NAD^+^ in liver tissues and L02 cells were detected by commercial kits (Nanjing Jiancheng Bioengineering Institute, Nanjing, China).

### *Detection of reactive oxygen species* (*ROS) production*

L02 cell suspensions were fixed on glass slides. Added 2 mL cell culture fluid and continue to culture for about 6 h. Added 1 mL dihydroethidium (Cat.No. GDP1018) which was dissolved in DMSO at a ratio of 1:1000 to each well and incubated in the dark. Added an appropriate amount of DAPI solution to the wells and stained. Then added a drop of anti-fluorescence quenching mounting plate into the hole, observed and took pictures under a fluorescent microscope (Olympus, Japan).

### Western blotting

Extracted proteins from cells and tissues as directed by the radioimmunoprecipitation assay (RIPA) kit (Sigma-Aldrich, USA). Added 5–10 μL of the collected protein samples to the SDS-PAGE gel sample holes. Primary antibodies against the following targets were used: SIRT1 (Cat.No. 9475, CST), NAMPT (Cat.No. 236874, Abcam), IDO (Cat.No. 13268-1-AP, Sanying), AMPK (Cat.No. 32047, Abcam), p-AMPK (Cat.No. 131357, Abcam) and GAPDH (Cat.No. 8245, Abcam). The intensity of the bands on the western blots was evaluated by Image Lab statistical software (Bio-Rad, USA).

### Statistical analyses

Each experiment was repeated at least three times and the data are expressed as the means ± SDs. Statistical analysis was performed using GraphPad Prism software version 8.0. Differences among multiple groups were evaluated using conventional Student’s *t* test or ANOVA followed by Tukey’s multiple comparison post hoc test (normally distributed data). Statistical significance was considered at *P* < 0.05.

## Results

### Fusobacterium* nucleatum* was abundant in liver tissues of ALF and linked to disease severity

**Fig. 1:**
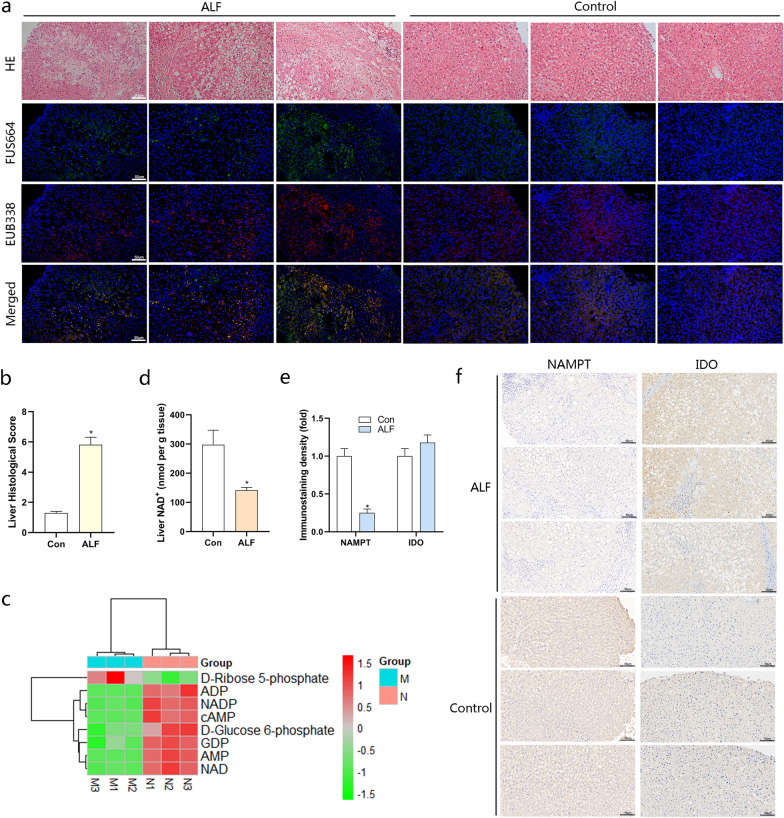
* Fusobacterium nucleatum* is Associated with ALF activity. **a** The representative images of HE staining and FISH of liver in each group to assess the amount of *F. nucleatum* in ALF and healthy control tissues. FUS664 (green) is a FITC-conjugated *F. nucleatum*-specific oligonucleotide probe; EUB338 (red) is a Cy3-conjugated universal bacterial oligonucleotide probe. Magnification, 200×. **b** The liver histological score of liver in each group (*P < 0.05; unpaired Student’s *t*-test; the error bars indicate the SDs). **c** Liquid Chromatography–Mass Spectrometry (LC–MS) Metabolomics of liver in each group. **d** NAD^+^ level in liver tissues obtained by hepatectomy from patients. **e**, **f** Immunohistochemistry analysis on the protein levels of NAMPT and IDO in liver tissues obtained by hepatectomy from patients. *P < 0.05; unpaired Student’s *t*-test; The error bars indicate the SDs for triplicate samples

To explore the potential relationship between *F. nucleatum* and ALF, we used FISH to detect the abundance of *F. nucleatum* in 15 liver tissues of ALF patients and 15 normal liver tissues. The liver histological score and the abundance of *F. nucleatum* detected in liver tissues of ALF patients was significantly higher than that of normal liver tissues (Fig. 1a, b). Then we analyzed the metabolic patterns of liver tissues of ALF patients and normal controls. Compared with the normal control group, the liver tissue of ALF patients showed inhibition of energy metabolism (Fig. 1c). As ATP and NAD^+^ have an excitatory effect on immune cells and adenosine has an anti-inflammatory effect on immune cells, NAD^+^ is an important coenzyme that mediates redox reactions [24]. We have detected that compared with normal liver tissue, liver NAD^+^ levels in ALF patients are significantly lower (Fig. 1d). According to reports, lack of NAD^+^ can cause moderate hepatic inflammation and damage [25]. We speculated that in the process of ALF, NAD^+^ biosynthesis was impaired. To this end, we tested the expression levels of major enzymes that control NAD^+^ biosynthesis, NAMPT and indolamine 2,3-dioxygenase (IDO) are rate-limiting enzymes for NAD^+^ salvage and de novo biosynthesis pathways respectively [26]. Interestingly, we found that the expression of NAMPT in liver tissues of ALF patients decreased. At the same time, the expression level of IDO had not changed much (Fig. 1e, f). We then evaluated the relationship between the abundance of *F. nucleatum* and clinicopathological features as shown in Table 1. The abundance of *F. nucleatum* was positively associated with the clinical course and refractory behavior (P < 0.05). These results showed that the NAD^+^ salvage synthesis pathway controlled by NAMPT might be an important factor in the consumption of NAD^+^ in the process of ALF, and the infection of *F. nucleatum* might be an important link, while the NAD^+^ de novo synthesis pathway might be an adaptive response.

**Table 1:** Clinicopathologic characteristics in *F. nucleatum*-negative vs. *F. nucleatum*-positive ALF

Characteristics	*F.nucleatum*-negative (n = 15)	*F.nucleatum*-positive(n = 15)	*p* value^a^
Gender			
Male	6	9	0.126
Female	9	6	
Age			
≤ 16	0	1	0.505
≤ 40	11	7	
> 40	4	7	
Clinical course			
Moderate	11	2	0.035*
Severe	2	13	
Remission	2	0	
PT activity (75% ~ 135%)	35% ± 5	20% ± 5	
Total bilirubin (0 ~ 23 μmol/L)	200 ± 30	350 ± 50	
Procalcitonin (< 0.1 ng/mL)	0.5 ± 0.2	0.8 ± 0.2	
HEV-IgG (0 ~ 1)	17 ± 3	14 ± 5	
AST (15 ~ 40U/L)	3150 ± 150	3850 ± 200	
ALT (9 ~ 50U/L)	3230 ± 300	3450 ± 150	
Behavior			
B1	7	5	0.495
B2	5	4	
B3	3	6	
Surgery			
Yes	3	11	0.017*
No	12	4	

^a^Chi-square test, *P < 0.05

### *Fusobacterium nucleatum* aggravated the degree of inflammatory damage in ALF model and regulated the expression of NAMPT

**Fig. 2:**
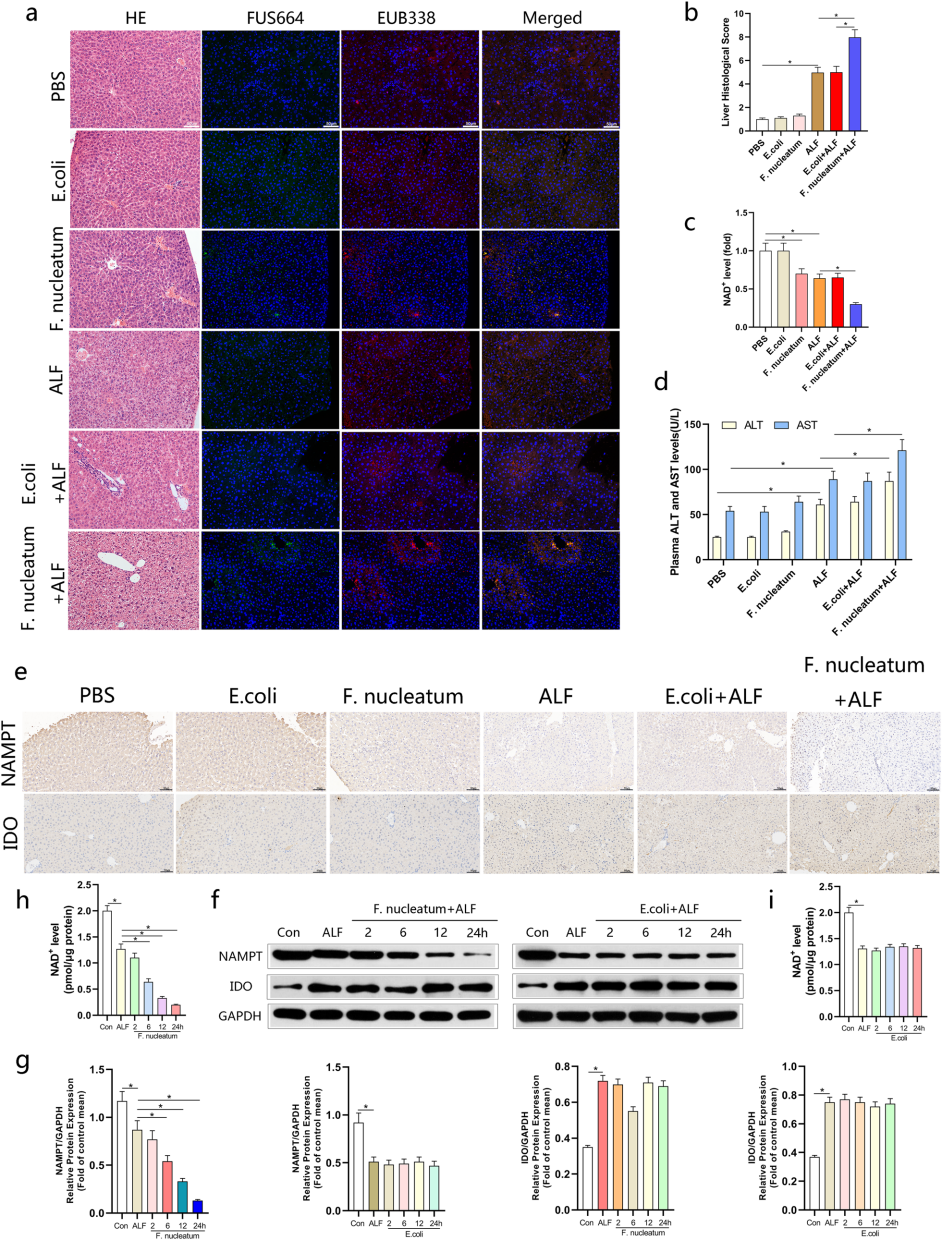
* Fusobacterium nucleatum* aggravated the degree of damage in ALF model and regulated the expression of NAMPT in vitro and in vivo*.* **a** Mice (n = 5–7 per group) were administered *F. nucleatum*, *E.coli* or PBS for 4 weeks and treated with LPS and D-gal for another 24 h. The representative images of FISH to assess the amount of *F. nucleatum* in livers of each group. Representative images of histological analyses are shown in (**a**) and quantified in (**b**) (200 × magnification). **c** NAD^+^ level in liver tissues obtained from mice. **d** The plasma levels of ALT and AST were measured in each group. **e** Immunohistochemistry analysis on the protein levels of NAMPT and IDO in liver tissues obtained from mice. **f**, **g** Western blotting was performed to measure the expression of NAMPT and IDO in L02 cells cocultured with *F. nucleatum*, *E. coli* or PBS (Control, Con) and quantified. Data shown are means ± SD of three separate experiments. *P < 0.05; one-way ANOVA combined with Bonferroni's post hoc test; the error bars indicate the SDs for triplicate samples. **h**, **i** The NAD.^+^ content of L02 cells treated by *F. nucleatum* or *E.coli* with a time gradient. *P < 0.05; unpaired Student’s *t*-test; The error bars indicate the SDs for triplicate samples

We hypothesized that the infection of *F. nucleatum* might aggravated the degree of inflammatory damage during ALF. To tested this hypothesis, we established the model of ALF. Compared with the normal control group, the mice treated with *F. nucleatum* or *E. coli* alone did not change significantly. However, mice treated with ALF + *F. nucleatum* exhibited more severe liver inflammation symptoms compared with the ALF group and *E. coli* + ALF group, including massive hemorrhagic necrosis, liver lobule structure disorder, and obvious inflammatory cell infiltration and higher histological scores (Fig. 2a, b). The 24 h survival rate of mice was observed in each group. The results showed that 40.0% of mice survived in *E. coli* + ALF group and ALF group, whereas only 16.0% in *F. nucleatum* + ALF group (Additional file 1: Fig. S1a). In addition, we evaluated the liver NAD^+^ levels and plasma levels of liver enzymes in each group. Consistent with previous observations, ALF mice showed lower NAD^+^ levels after the pretreatment of *F. nucleatum* (Fig. 2c)*.* The levels of ALT and AST in the ALF group increased significantly and *F. nucleatum* treatment could significantly aggravate this abnormal increase (Fig. 2d). In addition, we tested the expression levels of NAMPT and IDO in vivo and in vitro respectively. Immunohistochemical staining showed that the expression level of NAMPT in mice interfered with *F. nucleatum* decreased, while that in ALF mice decreased significantly, and the expression level decreased further after the intervention of *F. nucleatum*. During this period, the expression level of IDO did not decrease but increased slightly (Fig. 2e). In vitro we incubated L02 cells with *F. nucleatum* or *E. coli* in a time-dependent manner. Western blotting results showed that the level of NAMPT was positively correlated with the time of *F. nucleatum* intervention, while the level of IDO did not change significantly. In addition, the intervention of *E. coli* had no effect on the expression of NAMPT and IDO (Fig. 2f, g). We also tested the level of NAD^+^ at the cellular level, and the results showed that with the intervention of *F. nucleatum*, the level of NAD^+^ gradually decreased, while the intervention of *E. coli* had no effect (Fig. 2h, i). These results showed that *F. nucleatum* might affect NAD^+^ by regulating the expression level of NAMPT, and further promote the progress of ALF.

### *Fusobacterium nucleatum* aggravated macrophages infiltration and pro-inflammatory response in ALF model

**Fig. 3:**
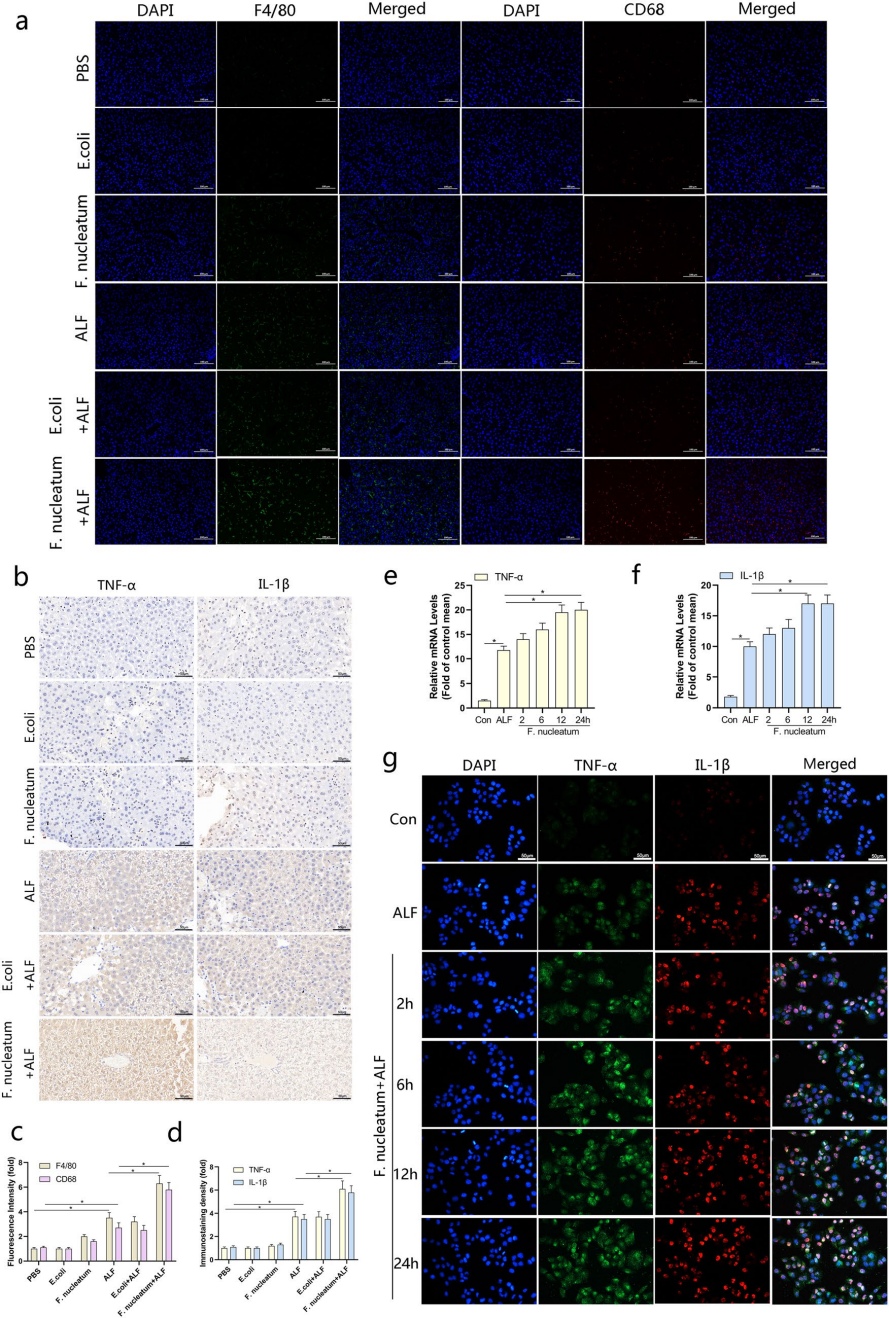
*Fusobacterium nucleatum* aggravated macrophages infiltration and pro-inflammatory response in vitro and in vivo. **a** Representative images and quantitative analysis (**c**) of infiltrated monocytes by F4/80 (left panel) and CD68 (right panel) immunofluorescence staining in liver tissues of each group. *P < 0.05. n = 6 for each group. **b** Immunohistochemistry analyses of the pro-inflammatory factors TNF-α and IL-1β in liver tissues and quantitative analysis (**d**). *P < 0.05. n = 6 for each group. (**e**–**g**) real-time PCR and immunofluorescence staining were performed to measure levels of pro-inflammatory cytokines in L02 cells infected by *F. nucleatum* with a time gradient. *P < 0.05 vs. Data were analysed by Student’s *t*-test. The error bars indicate the SDs for triplicate samples

Since endotoxins derived from the intestine can not only directly destroy liver tissues, but also induce local non-specific hypersensitivity reactions in the liver, induce macrophages to release a large amount of cytokines, and further produce natural immune cascades, resulting in a "second blow", and aggravated the damage of liver cells in the process of ALF. Therefore, we tested the level of macrophages infiltration and the degree of inflammatory response. As showed in Fig. 3a, the result of immunofluorescence revealed that the number of macrophages within liver tissues was increased in ALF model and infection with *F. nucleatum* enhanced this effect, but there was no similar macrophages infiltration in *E. coli* pretreated mice (Fig. 3a, c). Besides, the expression of TNF-α, and IL-1β in the liver tissues of ALF model were up-regulated and pretreatment with *F. nucleatum* enhanced this effect. At the same time, the structural damage of the liver lobules was more obvious and *E.coli* pretreated mice had no such effect (Fig. 3b, d). We found the same phenomenon in vitro. As the infection time of *F. nucleatum* increased, the expression levels of TNF-α and IL-1β also increased (Fig. 3e-g). Not only that, the protein level of anti-apoptotic protein Bcl-2 decreased, and the level of pro-apoptotic protein Bax increased (Additional file 1: Fig. S1c). These results indicated that the infection of *F. nucleatum* might aggravate macrophages infiltration and inflammation in the ALF model, and promote hepatocyte apoptosis.

### *Fusobacterium nucleatum* inhibited the antioxidant capacity of the ALF model

**Fig. 4:**
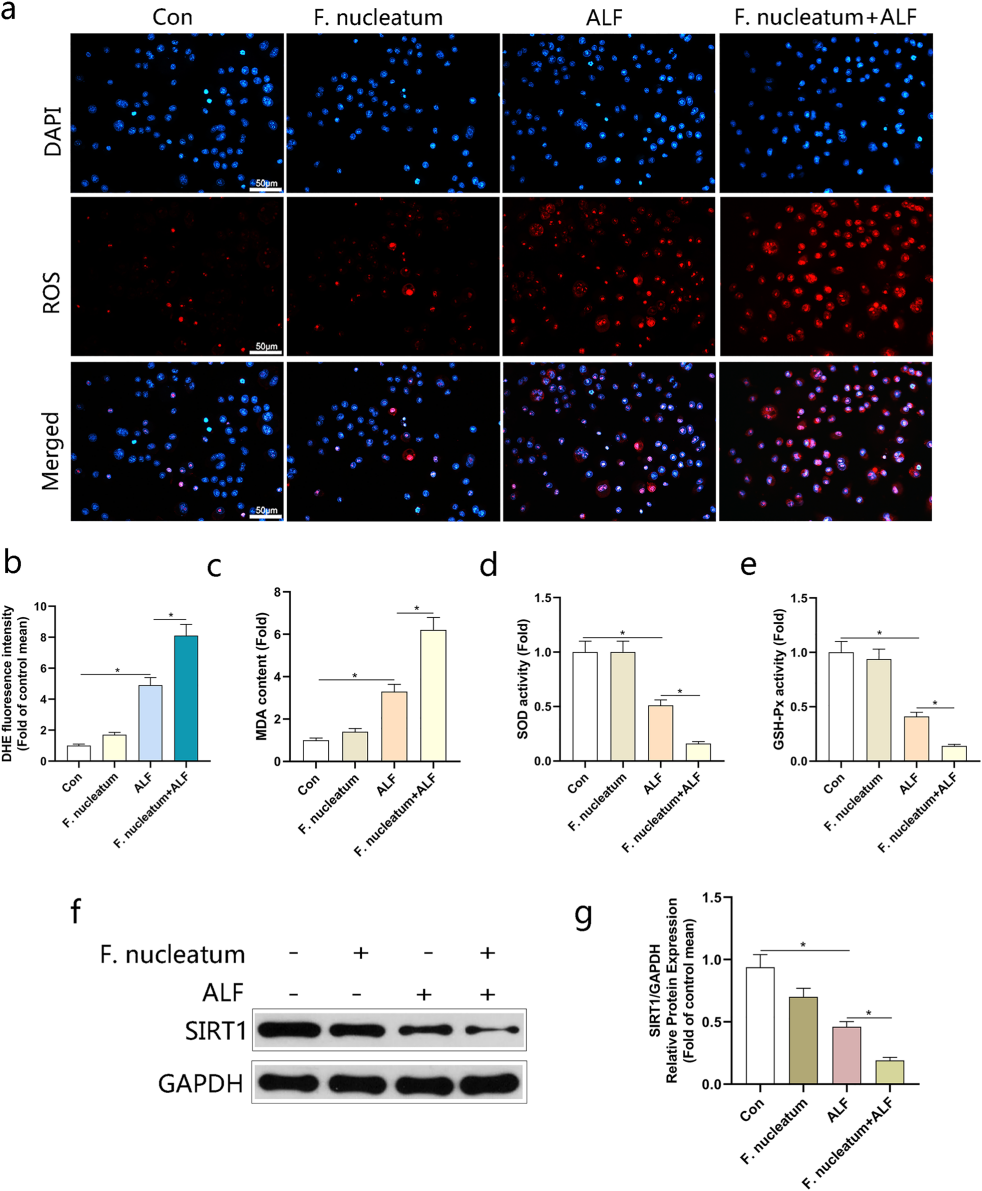
*Fusobacterium nucleatum* inhibited the antioxidant capacity of L02 cells. **a** ROS productions were detected by DHE staining. Representative images of the DHE staining in different groups. **b** ROS productions were evaluated by quantification of mean fluorescence intensity in DHE staining. **c**–**e** Levels of Malondialdehyde (MDA), Superoxide dismutase (SOD) and Glutathione peroxidase (GSH‐Px) in L02 cells. **f** Immunoblotting analysis on the protein levels of SIRT1 and quantified in (**g**). L02 cells stimulated with TNF-α (100 ng/mL) and D-Gal (44 μg/mL) were treated with *F. nucleatum* for 24 h. Data shown are means ± SD of three separate experiments. *P < 0.05; one-way ANOVA combined with Bonferroni's post hoc test; the error bars indicate the SDs

Next, we investigated the molecular mechanisms of the pro-inflammatory effect of *F. nucleatum* on the ALF model. Oxidative stress has been shown to promote inflammation during ALF [27]. Therefore, we evaluated the antioxidant capacity of the ALF model 24 h after the infection of *F. nucleatum.* As showed in Fig. 4a, b, the level of ROS stimulated in ALF model was significantly enhanced by *F. nucleatum.* Compared to the control group, the activities of malondialdehyde (MDA) were increased with the intervention of *F. nucleatum*, while superoxide dismutase (SOD) and glutathione peroxidase (GSH‐Px) were opposite, indicating that the antioxidant activity was decreased (Fig. 4c-e). The involvement of SIRT1 in the process of anti-oxidation and anti-inflammatory is well known. According to reports, NAD^+^ can affect the activity of SIRT1 and its expression level [28]. Our experiments showed that the expression of SIRT1 in the ALF model was reduced, and further decreased after the infection of *F. nucleatum* (Fig. 4f, g)*.* These results showed that *F. nucleatum* might have the ability to inhibit the antioxidant capacity in the ALF model.

### NAD^+^ supplementation reversed the inhibition of *F. nucleatum* on the antioxidant capacity of ALF models

**Fig. 5:**
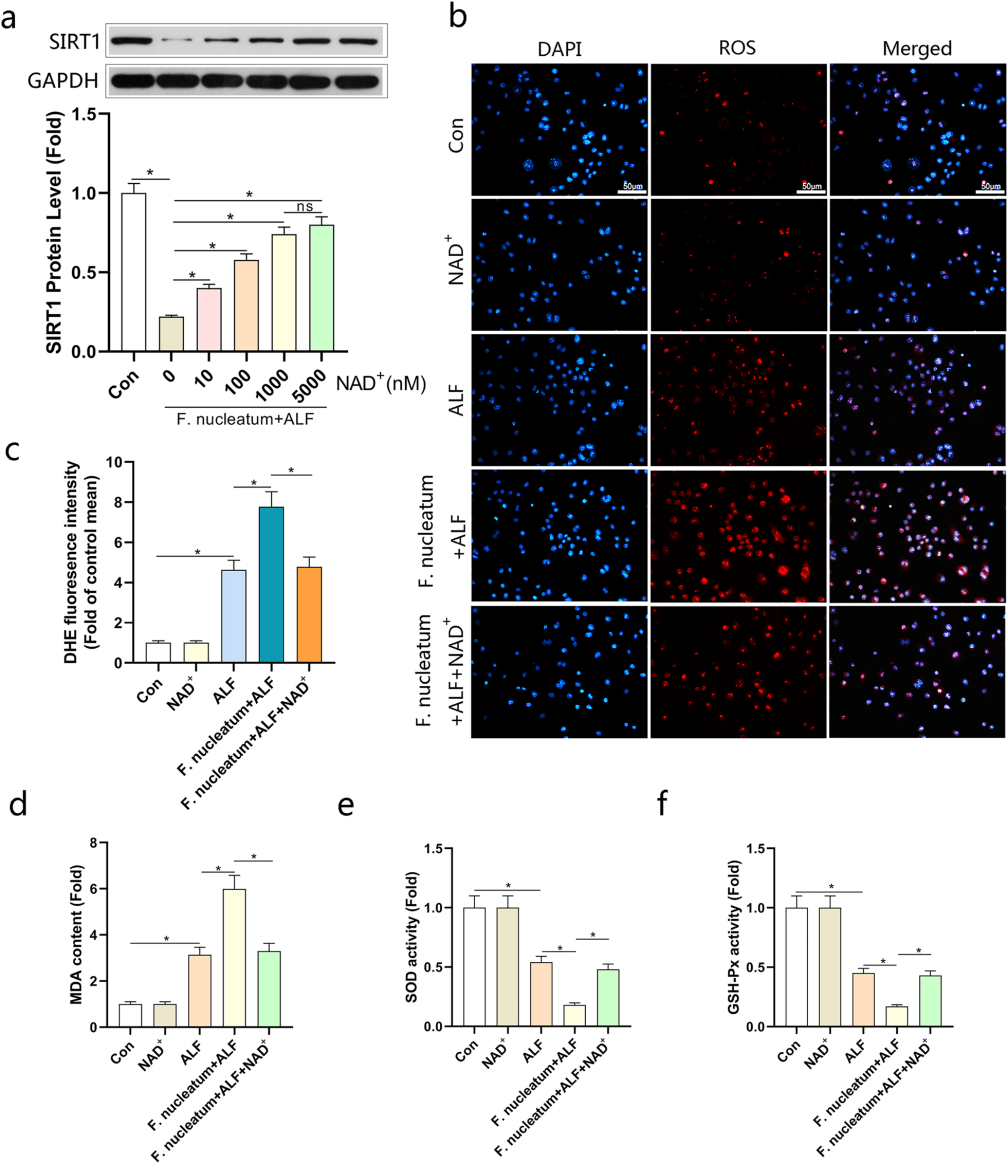
NAD^+^ supplementation reversed the inhibition of *F. nucleatum* on the antioxidant capacity of L02 cells. **a** Immunoblotting analysis on the protein levels of SIRT1 in L02 cells treated with different concentrations of NAD.^+^ and stimulated with TNF-α (100 ng/mL), D-Gal (44 μg/mL) and *F. nucleatum* for 24 h. **b** ROS productions were detected by DHE staining. Representative images of the DHE staining in different groups. **c** ROS productions were evaluated by quantification of mean fluorescence intensity in DHE staining. **d**–**f** Levels of Malondialdehyde (MDA), Superoxide dismutase (SOD) and Glutathione peroxidase (GSH‐Px) in L02 cells. Data shown are means ± SD of three separate experiments. *P < 0.05; one-way ANOVA combined with Bonferroni's post hoc test; the error bars indicate the SDs

Since SIRT1 is the most studied NAD^+^-dependent effector [29], we measured SIRT1 expression in L02 cells stimulated with *F. nucleatum*, as showed in Fig. 5a, administration of different concentrations of NAD^+^ in L02 cells could increase the expression of SIRT1 in a concentration-dependent manner even with the intervention of *F. nucleatum*, but the effect was not so obvious when the NAD^+^ concentration exceeds 1000 nM. Next, we used 1000 nM of NAD^+^ to intervene in L02 cells and detected the level of ROS production. Consistent with the previous results, NAD^+^ intervention could alleviate the generation of ROS in L02 cells stimulated by *F. nucleatum* (Fig. 5b, c). Besides, the activities of SOD and GSH‐Px were significantly enhanced with the intervention of NAD^+^, while MDA levels were reduced (Fig. 5d-f). These results showed that the supplementation of NAD^+^ might reverse the inhibition of *F. nucleatum* on the antioxidant capacity of ALF models.

### Supplemented the natural NAD^+^ precursor NR corrected the progression of ALF induced by *F. nucleatum* infection significantly instead of overexpression of SIRT1

**Fig. 6:**
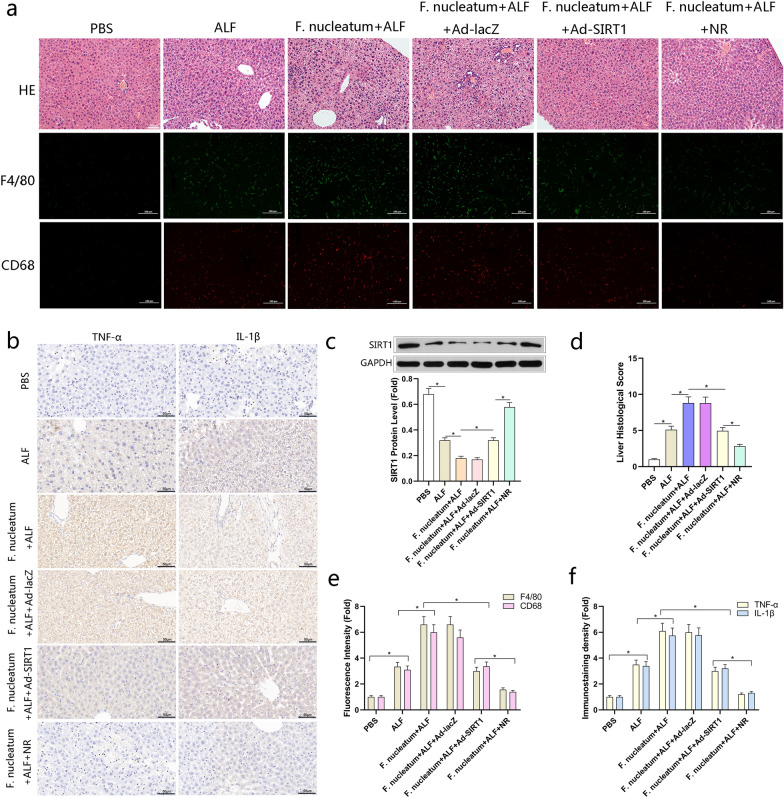
NR replenishment, but not SIRT1 overexpression, completely corrected the progression of ALF induced by *F. nucleatum* infection. **a** Representative images of HE staining and infiltrated monocytes by F4/80 and CD68 immunofluorescence staining in liver tissues of each group and quantified in (**d**, **e**). *P < 0.05. n = 6 for each group. **b** Effect of SIRT1 overexpression or NR on expression of the pro-inflammatory factors TNF-α and IL-1β in liver tissues and quantified in (**f**). *P < 0.05. n = 6 for each group. **c** Western blotting was performed to measure the expression of SIRT1 in liver tissues treated with SIRT1 overexpression or NR in mice infected with *F. nucleatum* and quantified. Data shown are means ± SD of three separate experiments. *P < 0.05; one-way ANOVA combined with Bonferroni's post hoc test; the error bars indicate the SDs

Next, we verified the effect of supplementing NAD^+^ on the ALF model of *F. nucleatum* infection in vivo. Mice were pretreated with *F. nucleatum* and NAD^+^ precursor NR, a widely-used NAD^+^ precursor for increasing NAD+ content [30]. Consistent with in vitro experimental results, compared with the ALF model of *F. nucleatum* infection, NR treatment could reduce the histological score of the ALF model (Fig. 6a, d), reduce the infiltration of macrophages (Fig. 6a, e) and the expression of inflammatory factors (Fig. 6b, f). The 24 h survival rate of mice was observed in each group. The results showed that 66.6% of mice survived in NR treatment group and ALF group, whereas only 16.0% in *F. nucleatum* + ALF group (Additional file 1: Fig. S1b). In addition, we investigated whether the loss of SIRT1 caused by NAD^+^ pool consumption could totally explain the susceptibility of *F. nucleatum* infection to the ALF model. In vivo, we injected the adenovirus carrying SIRT1 (Ad-SIRT1) from the tail vein to increase the protein expression level of SIRT1 in the liver of mice in the ALF model (Fig. 6c). As showed, SIRT1 overexpression significantly reversed the histological score in the ALF model and 33.3% of mice survived (Additional file 1: Fig. S1b), but the effect was not as obvious as the NR pretreatment group (Fig. 6a, d). The results of immunofluorescence and immunohistochemistry also demonstrated that SIRT1 overexpression could inhibit macrophages infiltration and the expression of inflammatory factors at a certain extent, but the effect was insufficient obviously compared with the NR pretreatment group (Fig. 6a-f). These results showed that the supplementation of NAD^+^ precursor NR corrected the progression of ALF induced by *F. nucleatum* infection significantly instead of overexpression of SIRT1.

### *Fusobacterium nucleatum* inhibited SIRT1 expression in a NAMPT/NAD^+^ dependent manner in the ALF model

**Fig. 7:**
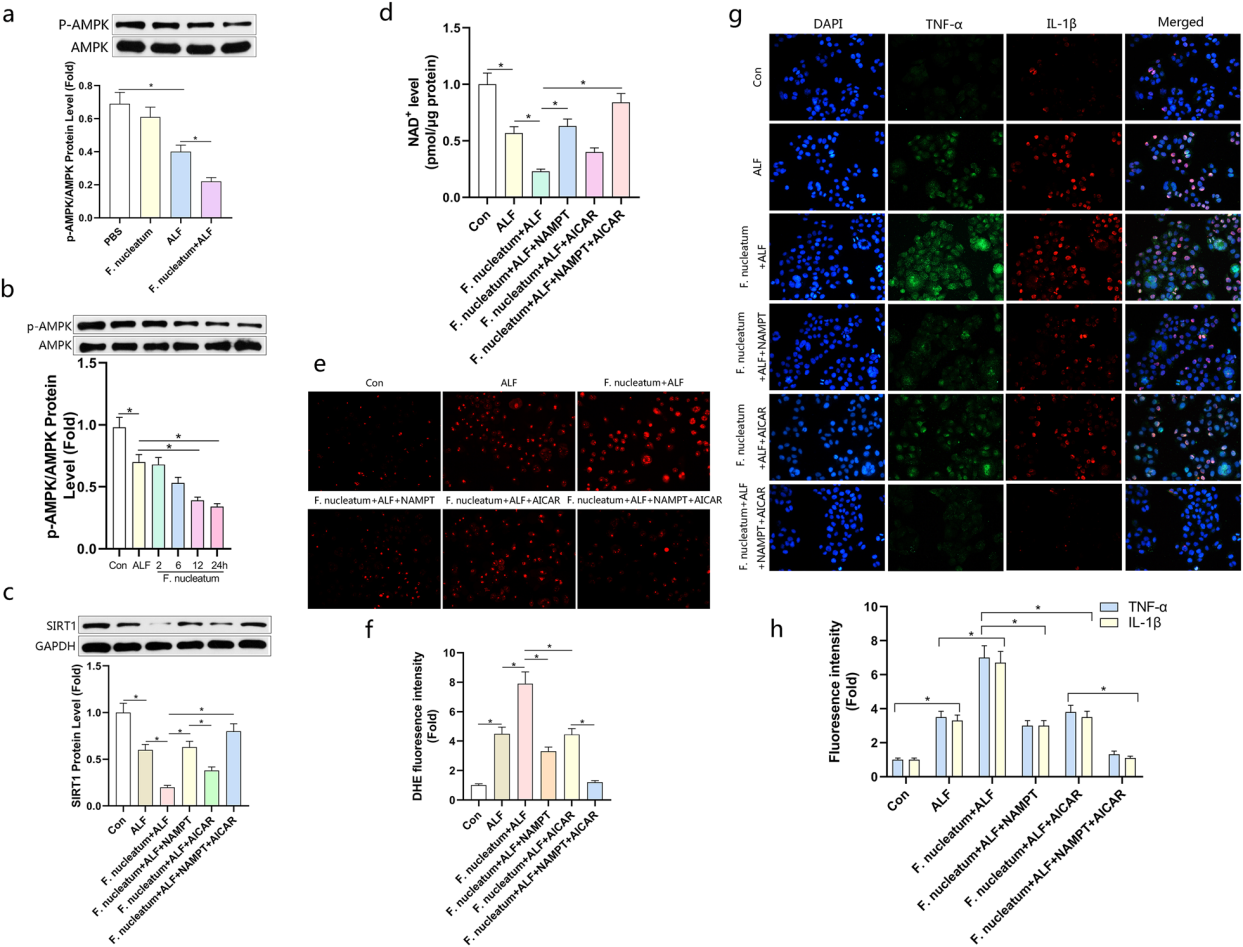
*Fusobacterium nucleatum* inhibited NAD^+^ via the salvage synthesis pathway of NAD^+^. **a** Western blots analysis of p-AMPK in mice treated with LPS (100 μg/kg) and D-gal (400 mg/kg) or infected with *F. nucleatum* and quantified. **b** Western blots analysis of p-AMPK in L02 cells stimulated with TNF-α (100 ng/mL), D-Gal (44 μg/mL) and infected by *F. nucleatum* with a time gradient and quantified. **c**, **d** The cells were transfected with NAMPT plasmid or treated with AICAR for 24 h and then stimulated with TNF-α (100 ng/mL), D-Gal (44 μg/mL) and infected by *F. nucleatum* for 24 h. Western blotting was performed to measure the expression of SIRT1 and the NAD^+^ content of cells were measured in each group and quantified. **e** Representative images of the DHE staining in different groups and evaluated by quantification of mean fluorescence intensity (**f**). **g** immunofluorescence staining were performed to measure levels of pro-inflammatory cytokines and quantified in (**h**). Data shown are means ± SD of three separate experiments. *P < 0.05; one-way ANOVA combined with Bonferroni's post hoc test; the error bars indicate the SDs

Many factors in the cell can regulate the content of NAD^+^ through different ways. Among the most important factors, besides NAMPT, AMPK increases the content of NAD^+^ by increasing mitochondrial β-oxidation [31]. Therefore, we tested the effect of *F. nucleatum* on the expression level of p-AMPK in the ALF model in vivo and in vitro. The results showed that the intervention of *F. nucleatum* could reduce the expression of p-AMPK indeed, and it gradually decreased in L02 cells in a time-dependent manner (Fig. 7a, b). Next, we wanted to figure out the main reason for the decrease of NAD^+^ and SIRT1 in *F. nucleatum* infection. We constructed the NAMPT overexpression plasmid and pretreated the cells with AMPK activator AICAR. As showed in Fig. 7c, western blotting results showed that the expression of SIRT1 was up-regulated after overexpression of NAMPT or activation of AMPK under the intervention of *F. nucleatum*, but the overexpression of NAMPT was significantly more effective than the activation of AMPK. In the case of supplementation of NAMPT, it was more effective after the activation of AMPK. We also found the same results in the NAD^+^ test (Fig. 7d). We continued to compare the levels of ROS in L02 cells after different treatments. Compared with activating AMPK, the overexpression of NAMPT could clear ROS in the ALF model infected by *F. nucleatum* significantly more effectively (Fig. 7e, f). In addition, immunofluorescence detected the expression level of inflammatory factors, and the inflammatory response after activating AMPK was still more severe than the overexpression of NAMPT, but the inflammatory response was significantly weaker when the overexpression of NAMPT combined with the activation of AMPK (Fig. 7g, h). These results showed that the inhibition of *F. nucleatum* on NAD^+^ and SIRT1 was mainly through the salvage synthesis pathway of NAD^+^. In addition, after treatment with metronidazole, Western bolt results showed that the protein level of SIRT1 increased, and the levels of Bax and inflammatory factors decreased, suggesting that the levels of inflammation and apoptosis decreased (Additional file 1: Fig. S1d).

## Discussion

Since the liver is an important organ for human nutrition and metabolism, patients with ALF are often accompanied by severe metabolism abnormalities and intestinal microecological imbalances [32]. In addition, intestinal endotoxins can induce intestinal endotoxemia through the "Gut-Liver axis" and further damage liver cells when the intestinal mucosal barrier is severely damaged [4, 33]. Endotoxin derived from the intestine can not only directly destroy liver tissues, but also induce local non-specific hypersensitivity reactions in the liver, induce hepatic macrophages to release a large number of cytokines, and further produce a natural immune cascade reaction, resulting in a "second blow", and aggravated the damage of liver cell during ALF [5, 32]. Liver failure is accompanied by severe abnormal energy metabolism. Intestinal endotoxemia damages the energy metabolism of liver cells and may be an important factor in the occurrence of liver failure [4]. Therefore, we tried to start with the microbiota that cause intestinal inflammation and destroy the intestinal mucosal barrier, and explored the relationship between microbiota and ALF.

There are multiple cases reported that *F. nucleatum* is involved in the progression of acute purulent liver abscesses [34–36]. Infections involving *F. nucleatum* are uncommon and can be serious, with many abscesses requiring surgery [37]. Among non-spore-forming anaerobes, *F. nucleatum* can cause clinically unique single-microbial infections, usually accompanied by severe sequelae [38, 39]. Although *F. nucleatum* can cause serious infections, it is still a rare pathogen in clinical practice. According to the results of previous studies, *F. nucleatum* may play an important role in the damage of the intestinal mucosal barrier function [40, 41]. Therefore, we have to think about the role of *F. nucleatum* in acute liver inflammation. In our research results, *F. nucleatum* might be an opportunistic risk factor for patients with ALF. In addition, we established the ALF model to evaluate the role of *F. nucleatum* in acute liver inflammation and we have confirmed that the enrichment of *F. nucleatum* in liver tissues of ALF would aggravate the severity of liver inflammation.

It has been confirmed that ALF patients are accompanied by severe metabolic dysfunction, and we found that ALF patients had a disorder of energy metabolism through metabolomics analysis. Studies have confirmed that *F. nucleatum* and glucose metabolism are related to colorectal cancer in mechanism, biology and clinical [42]. However, there are few reports on the relationship between *F. nucleatum* and energy metabolism during ALF. Next, we started from the perspective of NAD^+^. It is an important coenzyme ubiquitous in biological metabolism, the center of energy metabolism, and an indispensable cofactor for mitochondrial oxidative phosphorylation. NAD^+^ can directly or indirectly affect many key cells functions, including oxidative phosphorylation and redox reaction, DNA repair and inflammation, cellular senescence and immune cell function [43]. We tested the level of NAD^+^ and the expression of key enzymes in the two main ways of NAD^+^ synthesis. We found that the level of NAD^+^ decreased during ALF, and the invasion of *F. nucleatum* aggravated this trend. Importantly, the invasion of *F. nucleatum* only affected the salvage synthesis pathway of NAD^+^, and had no obvious effect on the de novo synthesis pathway. In addition, since endotoxin from the intestine can not only directly destroy liver tissues, but also induce local non-specific hypersensitivity reactions in the liver and induce hepatic macrophages to release a large amount of cytokines. Therefore, we also tested the the influence of invasion of *F. nucleatum* on macrophage infiltration and pro-inflammatory response during ALF, the results showed that the invasion of *F. nucleatum* gradually aggravated the inflammatory response during ALF in a time-dependent manner and recruited more macrophages. Besides, ALF is often accompanied by hypoxia, ROS and the production of pro-inflammatory cytokines [44]. ROS increase with aging and are closely related to various diseases. The pro-inflammatory response mediated by ROS in macrophages is considered to be harmful in sepsis [45]. Therefore, it is necessary to detect whether the invasion of *F. nucleatum* has an impact on the production of ROS. As expected, the invasion of *F. nucleatum* increased the generation of ROS in the ALF model and significantly weakened the antioxidant capacity of liver cells. Accompanying the decrease of antioxidant capacity was the activity of SIRT1, which is a NAD^+^-dependent deacetylase. According to reports, SIRT1 is involved in the process of anti-oxidation and anti-inflammatory [46]. In general, *F. nucleatum* inhibited the production of NAD^+^ and the activity of SIRT1, leading to more serious oxidative damage during ALF. Therefore, we speculated that *F. nucleatum* might promote the progress of ALF and aggravate redox reaction by regulating the salvage synthesis pathway of NAD^+^.

Among the sirtuin deacetylases that consume NAD^+^, SIRT1 is considered to be the best factor regulating metabolism and inflammation [47]. However, we found that under the invasion of *F. nucleatum*, although SIRT1 overexpression partially prevented the liver inflammation in ALF mice, it failed to correct all the pathogenic effects, supplementation with NAD^+^ precursor seemed to be completely effective for liver inflammation in mice. These results suggested that other NAD^+^ depletion factors might also be involved in the progression of ALF besides SIRT1. Of course, this needs to be further confirmed.

It is reported that AMPK can increase NAD^+^ content by increasing mitochondrial β-oxidation [48]. In order to study whether *F. nucleatum* only affected the production of NAD^+^ through the salvage synthesis pathway, we compared NAMPT and p-AMPK. In our study, it was indeed observed that *F. nucleatum* infection reduced the activity of AMPK, but in follow-up experiments, we found that even if AMPK was activated, the recovery level of NAD^+^ was not as obvious as the overexpression of NAMPT, and the detection of SIRT1 also showed the same results. Not only that, we tested the antioxidant capacity and inflammatory response of liver cells again, and got the same results as before. Perhaps the regulation of *F. nucleatum* on NAD^+^ and SIRT1 was dependent on the NAMPT/NAD^+^ pathway.

Our research has significant clinical significance. Our work emphasizes the role of *F. nucleatum* in the progression of ALF and the therapeutic value of NAD^+^. In fact, other NAD^+^ substrates, such as nicotinamide, has also been shown to improve liver function [49]. Therefore, assessing the microbial population and restoring the NAD^+^ pool seems to provide a new way to improve inflammation. Of course, the potential clinical evaluations on more microbiota and other NAD^+^ substrates are necessary. In addition, our study may have a limitation: We did not collect more patient specimens, the amount of liver transplantation was too small. The limitation might cause errors in our research results. Nevertheless, these data on the invasion of *F. nucleatum* and the decline of NAD^+^ in ALF patients are still very interesting. Of course, this needs to be confirmed in more patients. In addition, *F. nucleatum* infections are usually treated with broad-spectrum β-lactam antibiotics and metronidazole. However, these antibiotics can negatively affect the normal flora. Therefore, it is of great significance to develop new narrow-spectrum antibiotics with anti-anaerobic activity.

## Conclusion

In summary, our research results indicate that NAD^+^ deficiency associated with *F. nucleatum* is a risk factor for the onset of ALF, and suggest that prevention of infection and supplementation of NAD^+^ may be a therapeutic strategy for the prevention and treatment of ALF.

## Supplementary Information

**Additional file 1:** **Fig. S1.** Effect of F. nucleatum and antibiotics on survival rate, inflammation and hepatocyte apoptosis in mice with ALF. **a**, **b** The 6 h, 12 h, 18 h, 24 h survival rates of mice in each group were observed. **c**, **d** The proteins expression of Bax, Bcl-2, TNF-α, IL-1β and SIRT1 were detected by western blotting. Data shown are means ±SD of three separate experiments. *P < 0.05; one-way ANOVA combined with Bonferroni's post hoc test; the error bars indicate the SDs.

## Data Availability

The original contributions presented in the study are included in the article. The datasets used and/or analysed during the current study are available from the corresponding author on reasonable request.

## Abbreviations

ALF: Acute liver failure
NAD^+^: Nicotinamide adenine dinucleotide
NAM: Nicotinamide
NMN: Nicotinamide mononucleotide
NAMPT: Nicotinamide phosphoribosyl transferase
*F.** nucleatum*: *Fusobacterium nucleatum*
IDO: Indolamine 2,3-dioxygenase
SIRT: Sirtuin
AMPK: AMP-activated protein kinase
AICAR: 5-Aminoimidazole-4-carboxamide-1-b-D-riboside
FISH: Fluorescence in situ hybridization
NR: Nicotinamide riboside
ALT: Alanine aminotransferase
AST: Aspartate aminotransferase
SOD: Superoxide dismutase
MDA: Malondialdehyde
GH-Px: Glutathione peroxidase

## References

[1] Stravitz RT, Lee WM (2019). Acute liver failure. Lancet.

[2] Wu M, Liao L, Jiang L (2019). Liver-targeted Nano-MitoPBN normalizes glucose metabolism by improving mitochondrial redox balance. Biomaterials.

[3] Rui L (2014). Energy metabolism in the liver. Compr Physiol.

[4] Tilg H, Cani PD, Mayer EA (2016). Gut microbiome and liver diseases. Gut.

[5] Krenkel O, Mossanen JC, Tacke F (2014). Immune mechanisms in acetaminophen-induced acute liver failure. Hepatobiliary Surg Nutr.

[6] Liu Z, Guo J, Sun H (2015). alpha-Lipoic acid attenuates LPS-induced liver injury by improving mitochondrial function in association with GR mitochondrial DNA occupancy. Biochimie.

[7] Lv H, Lv G, Chen C (2021). NAD(+) Metabolism maintains inducible PD-L1 expression to drive tumor immune evasion. Cell Metab.

[8] Shats I, Williams JG, Liu J (2020). Bacteria boost mammalian host nad metabolism by engaging the deamidated biosynthesis pathway. Cell Metab.

[9] Meng Y, Ren Z, Xu F (2018). nicotinamide promotes cell survival and differentiation as kinase inhibitor in human pluripotent stem cells. Stem Cell Rep.

[10] Lautrup S, Sinclair DA, Mattson MP (2019). NAD(+) in brain aging and neurodegenerative disorders. Cell Metab.

[11] Rajman L, Chwalek K, Sinclair DA (2018). Therapeutic potential of NAD-Boosting molecules: the in vivo evidence. Cell Metab.

[12] Yoshino J, Mills KF, Yoon MJ (2011). Nicotinamide mononucleotide, a key NAD(+) intermediate, treats the pathophysiology of diet- and age-induced diabetes in mice. Cell Metab.

[13] He C, Wang H, Liao WD (2019). Characteristics of mucosa-associated gut microbiota during treatment in Crohn's disease. World J Gastroenterol.

[14] Nishikawa J, Kudo T, Sakata S (2009). Diversity of mucosa-associated microbiota in active and inactive ulcerative colitis. Scand J Gastroenterol.

[15] Kostic AD, Chun E, Robertson L (2013). Fusobacterium nucleatum potentiates intestinal tumorigenesis and modulates the tumor-immune microenvironment. Cell Host Microbe.

[16] Actis GC, Pellicano R, Rosina F (2014). Inflammatory bowel diseases: current problems and future tasks. World J Gastrointest Pharmacol Ther.

[17] Malluta EF, Maluf-Filho F, Leite A (2019). Pancreatic endosonographic findings and clinical correlation in Crohn's disease. Clinics (Sao Paulo).

[18] Yu J, Chen Y, Fu X (2016). Invasive Fusobacterium nucleatum may play a role in the carcinogenesis of proximal colon cancer through the serrated neoplasia pathway. Int J Cancer.

[19] Cao P, Chen Y, Guo X (2020). *Fusobacterium nucleatum* activates endoplasmic reticulum stress to promote Crohn's disease development via the upregulation of CARD3 expression. FRONT PHARMACOL.

[20] Hu L, Liu Y, Kong X (2021). Fusobacterium nucleatum facilitates M2 macrophage polarization and colorectal carcinoma progression by activating TLR4/NF-kappaB/S100A9 cascade. Front Immunol.

[21] Canto C, Houtkooper RH, Pirinen E (2012). The NAD(+) precursor nicotinamide riboside enhances oxidative metabolism and protects against high-fat diet-induced obesity. Cell Metab.

[22] Wang Y, Chen Q, Jiao F (2021). Histone deacetylase 2 regulates ULK1 mediated pyroptosis during acute liver failure by the K68 acetylation site. Cell Death Dis.

[23] Siegmund B, Lear-Kaul KC, Faggioni R (2002). Leptin deficiency, not obesity, protects mice from Con A-induced hepatitis. Eur J Immunol.

[24] Linden J, Koch-Nolte F, Dahl G (2019). Purine release, metabolism, and signaling in the inflammatory response. Annu Rev Immunol.

[25] Zhou CC, Yang Xi, Hua X (2016). Hepatic NAD(+) deficiency as a therapeutic target for non-alcoholic fatty liver disease in ageing. Brit J Pharmacol.

[26] Ruggieri S, Orsomando G, Sorci L (2015). Regulation of NAD biosynthetic enzymes modulates NAD-sensing processes to shape mammalian cell physiology under varying biological cues. Biochim Biophys Acta.

[27] Tian Z, Chen Y, Yao N (2018). Role of mitophagy regulation by ROS in hepatic stellate cells during acute liver failure. Am J Physiol Gastrointest Liver Physiol.

[28] Revollo JR, Li X (2013). The ways and means that fine tune Sirt1 activity. Trends Biochem Sci.

[29] Canto C, Menzies KJ, Auwerx J (2015). NAD(+) metabolism and the control of energy homeostasis: a balancing act between mitochondria and the nucleus. Cell Metab.

[30] Mouchiroud L, Houtkooper RH, Moullan N (2013). The NAD(+)/sirtuin pathway modulates longevity through activation of mitochondrial UPR and FOXO signaling. Cell.

[31] Katsyuba E, Romani M, Hofer D (2020). NAD(+) homeostasis in health and disease. Nat Metab.

[32] Wang LK, Wang LW, Li X (2013). Ethyl pyruvate prevents inflammatory factors release and decreases intestinal permeability in rats with D-galactosamine-induced acute liver failure. Hepatobiliary Pancreat Dis Int.

[33] Tripathi A, Debelius J, Brenner DA (2018). The gut-liver axis and the intersection with the microbiome. Nat Rev Gastroenterol Hepatol.

[34] Candoni A, Fili C, Trevisan R (2003). Fusobacterium nucleatum: a rare cause of bacteremia in neutropenic patients with leukemia and lymphoma. Clin Microbiol Infect.

[35] Nagpal SJ, Mukhija D, Patel P (2015). Fusobacterium nucleatum: a rare cause of pyogenic liver abscess. Springerplus.

[36] Jayasimhan D, Wu L, Huggan P (2017). Fusobacterial liver abscess: a case report and review of the literature. Bmc Infect Dis.

[37] Denes E, Barraud O (2016). Fusobacterium nucleatum infections: clinical spectrum and bacteriological features of 78 cases. Infection.

[38] Riordan T (2007). Human infection with Fusobacterium necrophorum (Necrobacillosis), with a focus on Lemierre's syndrome. Clin Microbiol Rev.

[39] Nygren D, Holm K (2020). Invasive infections with Fusobacterium necrophorum including Lemierre's syndrome: an 8-year Swedish nationwide retrospective study. Clin Microbiol Infect.

[40] Chen Y, Chen Y, Cao P (2020). Fusobacterium nucleatum facilitates ulcerative colitis through activating IL-17F signaling to NF-kappaB via the upregulation of CARD3 expression. J Pathol.

[41] Su W, Chen Y, Cao P (2020). Fusobacterium nucleatum promotes the development of ulcerative colitis by inducing the autophagic cell death of intestinal epithelial. Front Cell Infect Microbiol.

[42] Hong J, Guo F, Lu SY (2020). *F. nucleatum* targets lncRNA ENO1-IT1 to promote glycolysis and oncogenesis in colorectal cancer. Gut.

[43] Covarrubias AJ, Perrone R, Grozio A (2021). NAD(+) metabolism and its roles in cellular processes during ageing. Nat Rev Mol Cell Biol.

[44] Tak E, Jung DH, Kim SH (2017). Protective role of hypoxia-inducible factor-1alpha-dependent CD39 and CD73 in fulminant acute liver failure. Toxicol Appl Pharmacol.

[45] Chen J, Lai J, Yang L (2016). Trimetazidine prevents macrophage-mediated septic myocardial dysfunction via activation of the histone deacetylase sirtuin 1. Br J Pharmacol.

[46] Liu TF, Vachharajani V, Millet P (2015). Sequential actions of SIRT1-RELB-SIRT3 coordinate nuclear-mitochondrial communication during immunometabolic adaptation to acute inflammation and sepsis. J Biol Chem.

[47] Imai S, Guarente L (2014). NAD+ and sirtuins in aging and disease. Trends Cell Biol.

[48] Canto C, Gerhart-Hines Z, Feige JN (2009). AMPK regulates energy expenditure by modulating NAD+ metabolism and SIRT1 activity. Nature.

[49] Varela-Rey M, Martínez-López N, Fernández-Ramos D (2010). Fatty liver and fibrosis in glycine N-methyltransferase knockout mice is prevented by nicotinamide. Hepatology (Baltimore, MD).

