# Supplementary figures and images for: Fusobacterium nucleatum promotes the development of acute liver failure by inhibiting the NAD+ salvage metabolic pathway
Source: Gut Pathog. 2022 Jun 28;14:29. doi: 10.1186/s13099-022-00503-2 (PMC9238040; doi:10.1186/s13099-022-00503-2)

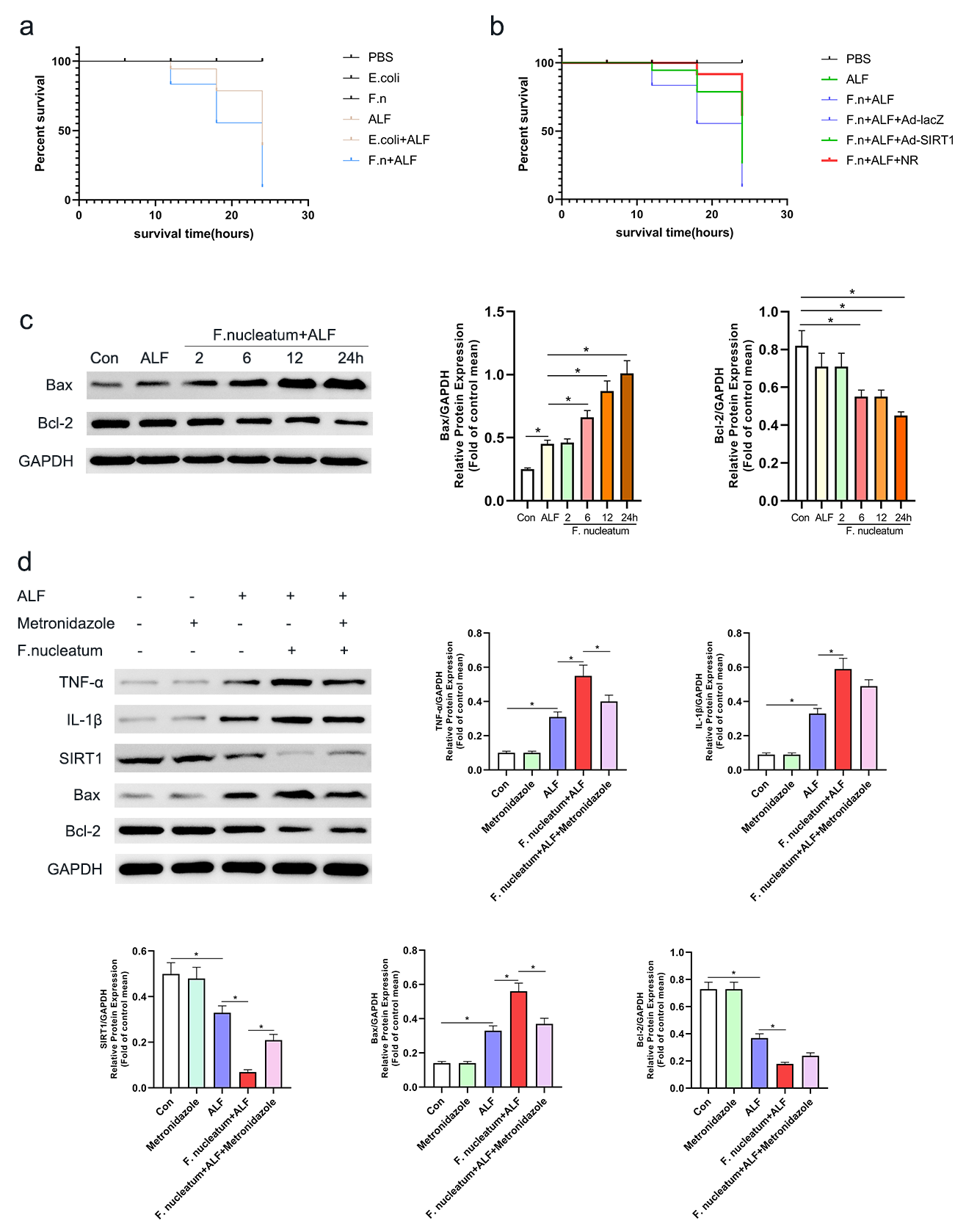

Supplement: Supplementary file 1 — Additional file 1: Fig. S1. Effect of F. nucleatum and antibiotics on survival rate, inflammation and hepatocyte apoptosis in mice with ALF. a, b The 6 h, 12 h, 18 h, 24 h survival rates of mice in each group were observed. c, d The proteins expression of Bax, Bcl-2, TNF-α, IL-1β and SIRT1 were detected by western blotting. Data shown are means ±SD of three separate experiments. *P < 0.05; one-way ANOVA combined with Bonferroni's post hoc test; the error bars indicate the SDs. [file 13099_2022_503_MOESM1_ESM.tif]
